# Indigenous students transitioning to school: responses to pre-foundational mathematics

**DOI:** 10.1186/2193-1801-3-685

**Published:** 2014-11-24

**Authors:** Grace Sarra, Bronwyn Ewing

**Affiliations:** Queensland University of Technology, Brisbane, Australia

## Abstract

Australian Indigenous students’ mathematics performance continues to be below that of non-Indigenous students. This occurs from the early years of school, due largely to knowledge and social differences on entry to formal schooling. This paper reports on a mathematics research project conducted in one Aboriginal community school in New South Wales, Australia. The project aimed to identify and explain the ways that young Australian Indigenous students (age 2–4 years) learn number language and processes, specifically attribute language, sorting, 1–1 correspondence and, counting. The project adopted a mixed methods approach. That is, the methodology was decolonising (Smith 1999) in that it collaborated with and gave benefit back to the Indigenous community and school being researched. It was qualitative and interpretative (Burns 2000) and incorporated an action-research teaching-experiment approach where and teachers collaborated with the researchers to try new teaching methods. This paper draws on data pertaining to students’ response to diagnostic interview questions, the pre- and post-test results of the interview and photographic evidence as observations during mathematics learning time. Participants referred to in this paper include one female principal (N = 1), and the transition class of students’ pre- (N = 6) and post-test (N = 3) results of the pre-foundational processes (also referred to as attributes). The results were encouraging with improvements in colour (34%), patterns (33%) and capacity (38%). As a result of this project, our epistemology regarding the importance of finding out about students’ pre-foundational knowledge and understandings and providing a culturally appropriate learning environment with resources has been built upon.

## Introduction

There is compelling international evidence that demonstrates participation in high quality early education and transition to school programs are particularly beneficial for children from disadvantaged backgrounds (see van Smeerdijk et al. 2014). The evidence indicates that participation enhances social ability and improvements in academic achievement in areas such as mathematics and language learning. Long term benefits include less likelihood of the need for special education, lower juvenile delinquency and, higher high school completion rates.

For Indigenous children, particularly those living in rural and remote Australia, equity of education in terms of access to high quality education is problematic (Sarra 2011). A range of challenges with the provision of education services are faced by children and families particularly those outside metropolitan regions. A lack of access to high quality education in the early years increases the likelihood of remedial education and limited future life opportunities.

Frigo and Adams (2002) identify many issues that exist for Indigenous students transitioning to school and argue that these are further perpetuated throughout school life:

In the early childhood years (0–8 years), Indigenous students are less likely to participate in pre-schooling that their non-Indigenous peers, they have higher rates of absenteeism beginning in primary school, and early indications show that, as a group, they perform at a lower level compared to their non-Indigenous peers (p. 1).

Findings in the 2011 census identified an increase in attendance at school with 56% of 3 to 5 year old Indigenous children attending pre-school or primary school. This rate is up from 53% in the 2006 census data (Australian Bureau of Statistics 2013).

Children learn most effectively when there is a partnership between parents and teachers, a sense of community between home and school and when they feel safe and valued (Dockett and Perry 2013). Studies (Woods 2004; Young-Loveridge 2011) have shown a positive relationship between parental involvement in their children’s schooling and numeracy achievement. Prior-to-school numeracy language and understandings need to develop as naturally as possible from children’s social contexts (Dockett and Perry 2013; Turunen and Dockett 2013).

### Readiness to learn mathematics in the early years

Dockett and Perry (2013) found that children from disadvantaged backgrounds attended formal schooling with the same readiness to learn as compared with children from less disadvantaged backgrounds. The difference between both groups depended on their engagement with early number language and processes involving understanding number sequence, identifying ordinal positioning and simple word problems (Young-Loveridge 2011).

A study by Anthony and Walshaw (2009) discussed the importance of providing young children with the opportunity to learn mathematics at this early age. This research found that the development of mathematics begins at birth as babies are immersed in a world of mathematics and includes such things as problem-solving, measurement, and spatial skills. This study is supported by Warren et al. (2011) who state that young children are capable of engaging with mathematical concepts at an early age, one reason being because children enter different contexts with a substantive amount of intuitive mathematical knowledge which serves as a base for future learning. Further, additional studies have shown that having a sound understanding of mathematics at a young age makes for greater mathematical achievement in the future (Bottia et al. 2014).

Stephen (2010) explored the benefits of child-centred learning and examined how children learn. This research identified that utilising this strategy allows the child the opportunity to decide what to do and how to spend their time, with ‘play’ being the learning medium. Similarly, the research by Miller et al. (2012) examined how children learn. This study argued that understanding how children learn needs to be included in the curriculum in combination with subject knowledge. This was because the ages 3–7 constitute an important phase in a child’s development. However a research study conducted by Edwards and Cutter-MacKenzie (2011) revealed that in recent years the concept of child-centred play has been critiqued as an insufficient pedagogical approach for supporting children’s knowledge and development.

Child-centred play is criticised for having too much of a focus on activities and not on outcomes, skills and understanding. In addition, another study explored the need for children’s interests to be taken into account within the framework of early childhood education (Hedges et al. 2011). This research stated that universally, to date, there is little agreement to the nature of the early school years curricula; however there is much literature advocating that curricula for children, birth to five years should incorporate children’s interests. It has been identified that tailoring of curricula to a child’s needs has more substance as opposed to a traditionally child-centred play environment, and research identified argued that although they are only children, they are actually competent learners. Warren et al. (2011) reinforced that play based teaching provides many children with opportunities to developing their understanding of mathematics, which is vitally important for all in the early years.

### Theoretical framework

A cognitive view of learning states that children are naturally curious and have an inherent drive to make sense of their environment, that is, they will naturally seek out patterns and relationships (Baroody and Ginsburg 1990). However, mathematics is also culturally-based (Saxe 1991) and represents the view of a particular class and background. Therefore, mathematics teaching is best seen as enculturation (Bishop 1988) which implies that Indigenous culture should have a powerful role in Indigenous mathematics learning. This view is in harmony with that of many researchers (Ewing et al. 2010; Ezeife 2002; Sarra et al. 2011) who argue that successful educational performance, motivation, and attendance are primarily linked to teaching that takes account of culture.

**Figure 1:**
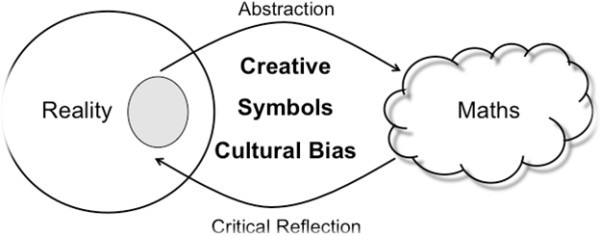
**RAMR framework.** According to Matthews (2008).

In the project a pedagogical model developed by researchers in the YuMi Deadly Centre (2012) was trialled because of its particular focus on teaching that was contextualised to students’ culture. The model was developed using a mathematical structure named RAMR which is an abbreviation for Reality, Abstraction, Mathematics and Reflection that contextualises the learning of mathematics to Aboriginal and Torres Strait Islander culture. The diagram in Figure 1 informed by the work of Ernest (1998) and Matthews (2008) encapsulates the framework by exploring the relationship and connection between culture and mathematics.

Mathematics starts from observations in a perceived reality. The observer chooses a particular part of the reality (represented by a grey circle), and then creates an abstract representation of the real-life situation using a range of mathematical symbols, which are put together to form a symbolic language we call mathematics. The observer uses the mathematics in its abstract form to explore particular attributes and behaviours of the real life situation and to communicate these ideas to others. From the mathematics, it is essential that the observer critically reflects on their mathematical representation to ensure that it fits with the observed reality. Consequently, the abstraction and critical reflection processes form an important cycle where mathematics and its knowledge are created, developed and refined (Matthews, p. 48).

Mathews identified three important features to the model in Figure 1 that needed to be emphasised when developing effective pedagogy in mathematics. First is creativity, which is particularly evident in the abstraction and critical reflection cycle. It is important to note that this cycle is similar to other artistic pursuits such as dance, music, painting and language as different forms of abstractions. Therefore, we can perceive mathematics as another art form and, in theory, relate it to these other forms of abstractions. In essence, it is possible to develop empowering pedagogy that allows students to be creative and express themselves in the mathematics classroom. This allows students to learn mathematics from their current knowledge (i.e., from the students’ social and cultural background), thereby providing agency through creativity and ownership over their learning. The abstraction process provides learning experiences using a variety of representations, actions and languages that enable meaning to be developed that carries mathematical ideas from reality to abstraction.

The second feature Matthews (2008) identifies is that as a product of the abstraction process, symbols and their meanings are important features of the model since they connect the abstract representation with reality. However, it is common that students do not make these connections easily and view mathematics as just sums with no real meaning. This is further exacerbated for students when they first learn algebra, and letters are suddenly introduced into mathematics without any obvious reason except that we are now learning algebra. Interestingly, focusing on creativity within mathematics, particularly with regard to the abstraction process, will naturally focus on symbols and meanings and assist in understanding the current mathematical symbols, and symbolic language, and their connection to the reality. This leads to the teaching and learning of the underlying structure of mathematics, providing students with a holistic view of mathematics.

The third important feature for developing pedagogy is to recognise cultural bias within mathematics. If we consider Figure 1, cultural bias exists in all aspects of the abstraction and critical reflection cycle. The observer expresses their cultural bias in the way they perceive reality and decide on which aspect of reality they wish to focus on. In the abstraction process, the form a symbol takes and the meanings that are attached to this symbol or group of symbols is biased by a cultural perspective.

Finally, the critical reflection processes are underpinned by the cultural bias within the abstraction process and the observer’s perception of reality. If we have an understanding and appreciation of the cultural bias within mathematics, new innovative pedagogy can be developed that moves beyond some cultural biases so that students can relate to mathematics and gain a deep understanding for the current form of mathematics and how mathematics is used.

## Methodology and method

The project adopted the empowering outcomes from of Smith’s (1999) decolonising methodology which aims to address Indigenous questions in ways that give sustained beneficial outcomes for Indigenous people. To meet the particular objectives of this study, the research was qualitative and interpretive (Burns 2000) and involved collaborative action-research (Kemmis and McTaggart 1988) where the research team work with leaders, teachers and teacher-aides and students at the community school playgroup and kindergarten to improve prior-to-school Indigenous children’s preparation for school in terms of number language and processes.

### The research site

Cabbage Tree Island Public School was established in an era of disturbing government policies and practices in Australia (Blake 2001 cited in Sarra 2008). The government at that time established Aboriginal reserves and missions with schools designed to control and discipline Aboriginal children (Blake 2001 cited in Sarra 2008). The overarching intention was to remove their freedom and use assimilation policies that abolished Indigenous knowledges, cultures and ways of doing and being from each Aboriginal child (Human Rights and Equal Opportunity Commission 1997).

From conversations and observations conducted during visits to Cabbage Tree Island by the research team, the school provided a culturally rich curriculum with strong links between the school and the community. This was evident through the significant contributions of elders and community members who shared their Aboriginal knowledges within the academic, social, political and cultural spheres of students learning experiences. These Aboriginal voices were valued in their roles of determining and identifying school priorities that were of importance to them to enable Aboriginal students to embrace a strong sense of their Aboriginality and provide opportunities that contributed to each child reaching their full potential as productive individuals of society.

### The Transition Program

Transition into Kindergarten was a yearlong process involving pre-school students reading, engaging in learning about mathematics, playing and following instructions in the K-1 classroom. In term 4 all students experienced blocks of learning in the K-1 room to build up to whole day experiences and consecutive days in preparation for Kindergarten in the following year.

### The participants

The Indigenous principal at the school had undertaken the Stronger Smarter Institute Leadership program (Sarra 2012) and implemented its vision and within her school community before we had commenced the project.

The children, referred to in this paper (N = 6) who attended the transition to school group at Cabbage Tree Island Public School were aged between 2–4 years and interacted closely with the Play Group. This group provided coordinated services to assist parents and their young children with transitioning to school and also to build relationships between parents, children and teachers. Students were known to staff prior to enrolment at the school to support students with additional services or to accelerate students to meet learning needs prior to Kindergarten. These students were taught by a non-Indigenous teacher and a full time Indigenous teacher aide who had been employed at the school for a number of years and resided in the local Indigenous community.

### Data collection strategies

In this paper the focus of data gathering pertains to students’ pre- and post-test results, their responses to the pre-test results, interview with Principal, and photographic evidence of classroom and school observations. The diagnostic interviews/tasks (Baturo 2008) were administered to the children in the transition year before commencing Kindergarten in New South Wales, Australia to probe their knowledge of pre-foundational processes and associated language.

**Figure 2:**
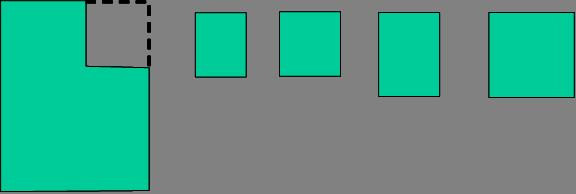
**Spatial visualisation.**

The diagnostic interview was designed to elicit students’ understanding of the important processes that are required for processing later mathematics. We wanted to find out what students knew before their entry to formal schooling and also to establish a starting point for supporting students with their learning using the RAMR model described previously. An example of the diagnostic questions is shown in Figure 2. It relates to spatial visualisation. This example is a representation of space, here, geometric shape. The task seeks to gain an understanding of the students’ capacity to visualise and demonstrate their spatial understanding using external representations. This task will be referred to in the analysis and discussion section.

## Analysis and discussion

In this section, we discuss the pre- and post-test results focusing on pre-foundational process also referred to as attributes, snapshots of students’ responses to tasks and snapshots of photographic observations taken during lessons.

### Pre- and Post-test results

**Figure 3:**
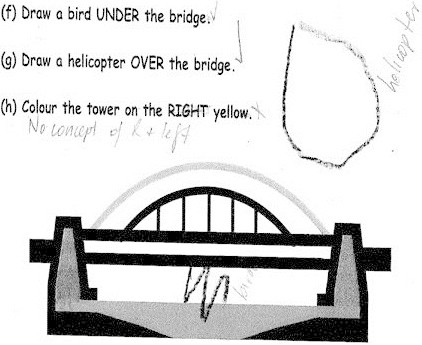
**Attribute check transition: pre- and post-test results.**

Transition students (N = 3) were pre- and post-tested on the following pre-foundational processes: colour, shape, visualisations, pattern, position, length, thickness, capacity and comparison. Results shown in Figure 3 indicate two students (N = 2) improved in the post-test while one student’s (N = 1) post-test results decreased. The class mean however demonstrates an overall improvement of 20%.

**Figure 4:**
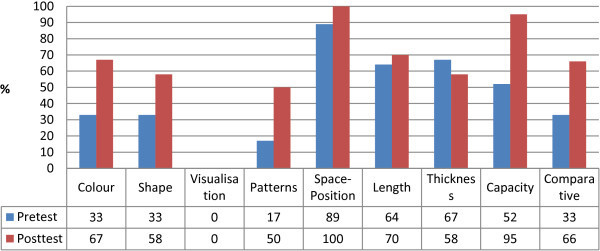
**Attributes check transition: Pre- and post-test.**

In Figure 4, the class mean demonstrates improved post-test results in all areas but visualisation and thickness. None of the three students was able to solve the visualisation task. Each of the students scored zero points out of one point in both pre- and post-tests.

In the thickness task the class mean suggests a decline in correct answers in the post-test. Only one student improved in this task. Another student achieved the same result in the pre- and post-test while the third student’s test result decreased.

Colour and patterns test results improved most significantly with an increase of 34% in colour and 33% in patterns. In colour, two student test results went up from 0% to 75% of correct answers in the post-test however one student scored 50% less than in the post-test (same student who worsened in the thickness task). In the patterns task, two students improved their results from 0% to 50% of correct answers while one student’s test result remained the same in the pre- and post-test.

One student’s results improved by 34% in space-position while the other two students achieved 100% of correct answers in both pre- and post-test. Whilst in the length task one student improved by 37% another student achieved the same result in the pre- and post-test. The same student whose results decreased in the thickness and colour task also worsened in the length post-test.

**Figure 5:**
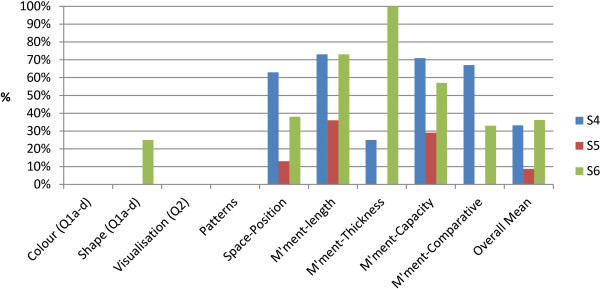
**Attribute check transition post-test only.**

Three students (N = 3) participated in the post-test only (Figure 5). No scores were achieved by the three students for the areas colour, visualisation and patterns.

Only one student scored 25% of correct answers in shape while the other two students also scored 0% in this area. The overall score for the post-test for these three students was lower (26%) compared to the pre-test of the students who participated in both pre- and post-test (43%).

### Snapshots of assessment

Accepting the notion that understanding is a dynamic process where learners move from the informal to the formal, we as teachers then must learn to recognise what learners know and understand about mathematics throughout the continuum of growth in learning (Bobis et al. 2009). As pointed out earlier in this paper, we need to take the time to find out and learn about children’s understanding of important mathematical concepts and processes that are needed for developing their mathematical knowledge and understanding further. In this section we provide snapshots of students’ thinking via pre- tests and photographic evidence. The examples are drawn from the research in this project.

**Figure 6:**
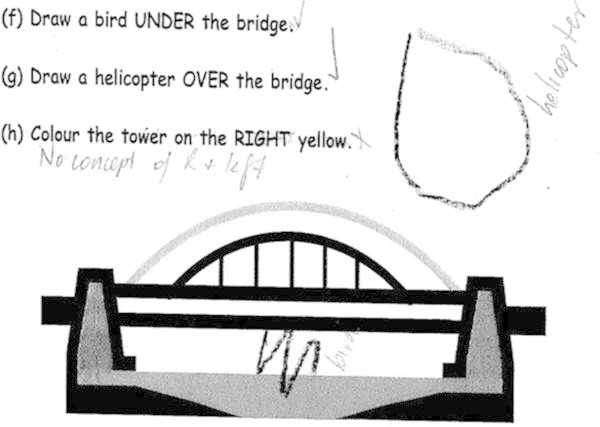
**Space and Position: Matthew’s response.**

A task widely used to gather knowledge about a student’s understanding of space and location is to ask the child to draw the location of different objects on a picture (Clements and Surama 2014). The researcher read the task shown in Figure 6 to the child.

Matthew’s response was his reaction to a previously unknown task. Therefore, it is informative with regards to what he knows about position and proximity such as under and over, right and left. He has drawn a bird under the bridge. The fact that he has drawn a shape to represent a helicopter is of no great concern since it does not interfere with his ability to show the idea of over. Matthew has no concept of right and left in this task. These two words can be the most difficult words when understanding position (Clements and Surama 2014). This difficulty increases when children are asked to differentiate right and left orientations on a worksheet image if they assume the orientation of the image. The words are a source of confusion for children for many years and usually not well understood until 6–8 years of age (Clements and Surama 2014).

**Figure 7:**
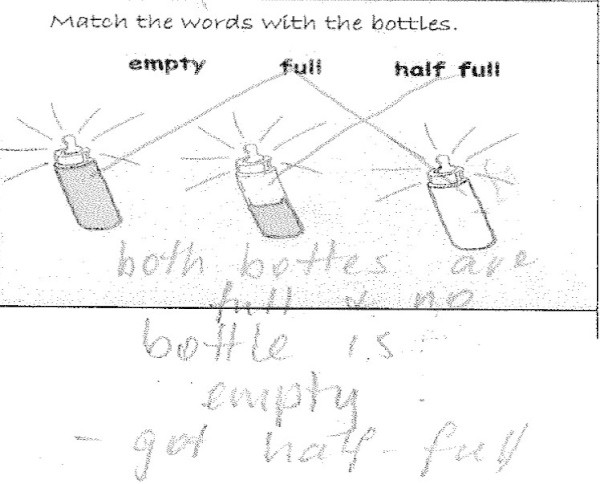
**Matching empty and full: Billie’s response.**

Another task that is used to gather information about children’s understandings of empty and full is to ask the child to match objects with different quantities to the words, empty, full and half full (Bobis et al. 2009). This task is shown in Figure 7.

Several important aspects critical to Billie’s understanding of empty, full and half full were identified in this task. He was able to match the words half full with the image of a half full bottle. He could match the full bottle with the word full by seeing the item, however Billie saw that two bottles were full stating “both bottles are full and no bottle is empty”. Whilst Billie was able to identify the “half” shaded area in the half full bottle, he was not able to differentiate which bottles was full and empty using the shading as a guide.

**Figure 8:**
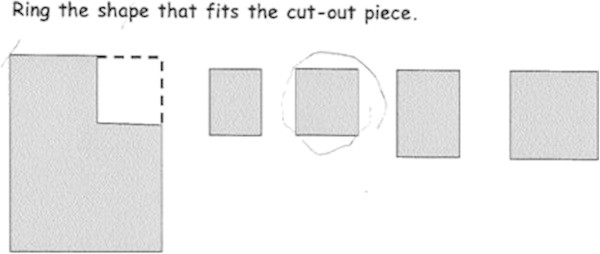
**Compare and match the shape: Marko’s response.**

In the next task, we wanted to find out what Marko understood about shape, in particular visualising and identifying the shape that fitted the cut-out piece. Marko was presented with the task shown in Figure 8 and asked to ring the shape that fitted.

We were concerned about whether Marko could visualise and demonstrate his understanding of shape using the representations provided. Further, we wanted to know if he could compare the four smaller shapes and make a decision about which one fitted the cut-out piece and distinguish between one shape and another. In the case of this task, Marko could. Distinguishing between shapes is one of the first levels of thinking through which children move as they construct their geometric thinking (Clements and Surama 2014).

### Snapshots of classroom observations

**Figure 9:**
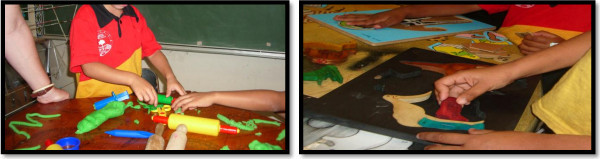
**Investigating length and shape.**

As part of the project we wanted to learn about the resources and environment where students were in transition and how they connected with the students’ every day experiences. We used observational photographic records as evidence of the resources used and the school environment as shown in Figure 9.

During transition lessons, the students were assisted with developing understandings of pre-foundational processes through the use of rhymes, stories, songs and Indigenous puzzles made by local Indigenous artists and crafts people. In the photographs above, the student on the left is experimenting with making long and short lengths using play dough. Play dough allows for experimentation of length where children can trial ideas and reshape previous ones. In the photograph on the right, the students are playing with the puzzles that provided experiences to assemble and re-assemble parts to make a whole or take away from.

**Figure 10:**
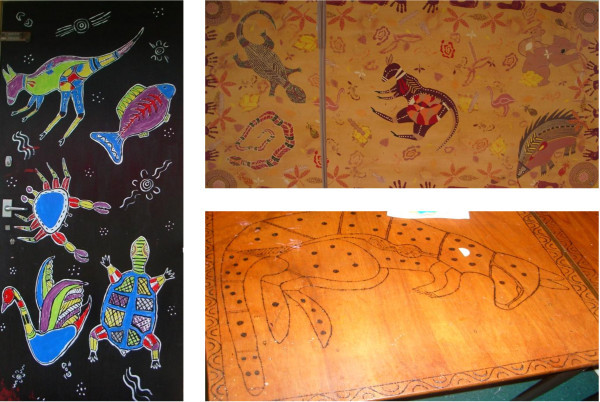
**Acknowledgement of a positive sense of Aboriginal identity.**

The next series of photographs shown in Figure 10, provide observational evidence pertaining to the schools’ acknowledgement of a positive sense of Aboriginal identity in and outside the classroom.

This acknowledgement was further enhanced with the engagement of the Indigenous community at Cabbage Tree Island Public through their weekly Bundjalung Language classes that focused on providing students with opportunities to learn their local Aboriginal language and about their Aboriginal culture to instill a positive cultural identity. The principal Dion Anderson (2013) stated, student behaviour and engagement is positive and interest is high. Our school signs and newsletters celebrate a positive cultural identity with the inclusion of Bundjalung language and in school murals designed by students and local Aboriginal artists.

The school has a dance group taught by a professional Aboriginal dancer who has developed students’ skills in dance but has explored story telling through expressive and creative dance. This has provided students with opportunities to reinforce their Aboriginal identity and continue to instil in them their sense of cultural pride.

## Conclusion

In this paper we have provided snapshots from a project that focused on young Indigenous children in transition to formal schooling, their pre-foundational mathematics knowledge and understanding, the learning and photographic observations of classroom resources and the school environment. In doing so, we have highlighted students’ knowledge and understanding of the pre-foundational processes on entry to transition in Term 4 of the school year and before exist from that term after a nine week teaching cycle. We found from the results presented that students overall made gains in their learning of these processes.

We also identified that the provision of culturally rich resources and learning environment developed students’ positive sense of Aboriginal identity which in turn developed a positive sense of self as a learner. The school in this project created such a learning environment for this to occur.

The RAMR model (YuMi Deadly Centre 2012) was trialled in the project because of its focus on high expectations for Indigenous learners and effective teaching practice in mathematics and with a view to enhancing transition students’ mathematical knowledge and understandings. Structured teaching lessons were modelled by the teacher and teacher-aide and drew on aspects of the RAMR model, namely, reality—contextualising to students’ culture and home language (Bishop 1988; Saxe 1991). Classroom observations recorded by the researchers demonstrated that displayed culturally appropriate resources encouraged the children to engage in the learning process and thus reducing the cultural, linguistic and contextual barriers in an out of the classroom setting. This was a critical element of the RAMR model.

In this education revolution, if we want educators to see Indigenous children as high calibre students and communities as highly engaged, then we must highlight and network such places. This way, it can be clearly observed, learned from, and emulated to the extent that this becomes the new reality of schooling for Australian Indigenous children.
